# Assessing Iron Status in Chronic Heart Failure Patients by Using Serum Ferritin and Transferrin Saturation Levels: A Cross-Sectional Descriptive Study

**DOI:** 10.7759/cureus.39425

**Published:** 2023-05-24

**Authors:** Uchenna M Amaechi, Henry O Aiwuyo, Chukwudum Ewelukwa, Nosakhare Ilerhunmwuwa, John O Osarenkhoe, Anthony G Kweki, Okoke Eseoghene Onuwaje, Amam Mbakwem, Michael Kehinde

**Affiliations:** 1 Internal Medicine, Lagos University Teaching Hospital, Lagos, NGA; 2 Internal Medicine, Brookdale University Hospital Medical Center, Brooklyn, USA; 3 Global Reseacher and Instructor, Love Your Menses, Inc., Boston, USA; 4 Medicine and Surgery, Igbinedion University Teaching Hospital, Benin City, NGA; 5 Internal Medicine/Cardiology, Colchester Hospital, East Suffolk and North Essex NHS Foundation Trust, Colchester, GBR; 6 Internal Medicine, Bowerfold, Blackburn, GBR; 7 Internal Medicine/Cardiology, College of Medicine, University of Lagos, Lagos, NGA; 8 Internal Medicine/Hematology, College of Medicine, University of Lagos, Lagos, NGA

**Keywords:** descriptive study, cross-sectional, transferrin saturation, serum ferritin, chronic heart failure, iron status

## Abstract

Background and objective

Chronic heart failure (HF) is a major medical condition worldwide and is associated with significant morbidity and mortality. Chronic HF could be complicated by iron deficiency (ID), and in severe cases, ID anemia, leading to negative HF outcomes even in people on optimal HF treatments. ID has been reported to be the most common nutritional deficiency in chronic HF. It is therefore important to study and analyze the relationship between these two variables. Identifying and treating the comorbidity of ID in chronic HF may help improve the treatment outcomes of chronic HF. In this study, we aimed to determine the iron status of chronic HF patients by using serum ferritin (SF) and transferrin saturation (TSAT).

Materials and methods

A cross-sectional descriptive study was conducted involving 88 Nigerian patients with chronic HF at the Lagos University Teaching Hospital (LUTH). The participants were evaluated based on their laboratory findings.

Results

ID was found in 34% of chronic HF patients. Of them, 17% had absolute ID while 17% had functional ID.

Conclusion

ID was present in about one-third of the chronic HF patients. It was more common and worse in patients belonging to advanced HF functional classes.

## Introduction

Iron is an important element, and one of the first five important micronutrients along with iodine, vitamin A, zinc, and folate [1]. It is particularly important for the body as it functions in the catalysis of several biochemical reactions apart from being a cofactor for many enzymes. It is important to note that iron within the body effectively shuttles between ferric and ferrous forms [2]. Iron is of utmost importance in the red blood cells as it functions as the major constituent for heme in the formation of hemoglobin (Hb), the major transporter of oxygen in the blood; it is also seen in myoglobin, which stores oxygen in the tissues and also crucial for several metabolic functions in the heart and skeletal muscle involving oxidative enzymes and respiratory chain proteins [3].

Heart failure (HF) is a clinical syndrome characterized by symptoms typically including breathlessness, ankle swelling, and fatigue that may be accompanied by signs such as elevated jugular venous pressure, pulmonary crackles, and peripheral edema and caused by a structural and/or functional cardiac abnormality, resulting in a reduced cardiac output and/or elevated intracardiac pressures at rest or during stress [4]. HF occurs in about 1-2% of the general adult population [5]. It accounts for about 2.21% of outpatients, 3-7% of general medicine admission in Sub-Saharan Africa, and 30% of hospital admission in specialized cardiovascular units in Africa [6,7]. In Western countries, its prevalence is high in individuals aged 50 years and above while a prevalence of 10% and above has been recorded among people older than 70 years [8]. Research studies have demonstrated that females exhibit a heightened frequency of the ailment due to their longer life expectancy, yet its incidence rate is greater among males [9,10].

Depending on the functional class, patients with chronic HF may suffer from severely reduced exercise capacity, manifesting as excessive breathlessness accompanied by fatigue. There is currently no conclusive evidence to suggest a direct correlation between these identified symptoms and the functionality of the myocardium. Nevertheless, these symptoms have been associated with a substantial decrease in quality of life and an increased incidence of morbidity [8]. Inadequate oxygen supply and impaired utilization of oxygen by exercising muscles, among many mechanisms, have been found to be inherent in chronic HF patients with exercise limitations [4,5]. Factors impairing oxygen supply or its utilization may thus be important in determining functional capacity and addressing these factors may have a positive impact on these chronic HF patients.

Recently, iron deficiency (ID) has been recognized as a cause of poor outcomes in patients with chronic HF despite standard anti-HF treatment. There is limited data describing the iron status of patients with chronic HF in the African setting and the presence of ID can worsen chronic HF severity among Africans [11,12]. In light of this, this study was carried out to determine the iron status in patients with chronic HF and provide evidence for future interventional trials with regard to iron therapy in African patients with chronic HF.

## Materials and methods

A cross-sectional descriptive study was carried out among adult patients with chronic HF attending cardiology outpatient clinics at the Lagos University Teaching Hospital (LUTH). LUTH is a tertiary hospital located in Idi-Araba, Surulere, Lagos State, Nigeria. The sample size was determined by using the formula for cross-sectional studies, n=(Z^2^pq)/d^2^ [13], and using a prevalence rate of ID of 26% as reported among chronic HF patients [14]. The sample size estimated was 95. The study was carried out for a period of six months. Participants were selected using the convenient sampling technique.

Interviewer-administered questionnaires were used to obtain sociodemographic characteristics and anthropometric measurements including weight and height. All participants underwent detailed clinical history and examination. Using Framingham criteria, clinical evidence of chronic HF was assessed in the participants [15]. Ethical approval was obtained from Lagos University Teaching Hospital Health Research Ethics Committee (HREC registration number: NHREC:19/12/2008a). Hematological parameters such as Hb concentration, mean corpuscular volume (MCV), mean corpuscular hemoglobin (MCH), mean corpuscular hemoglobin concentration (MCHC), and total WBC count were assessed in these patients. The iron status was determined by quantification of the serum iron, serum ferritin (SF), and transferrin saturation (TSAT). Serum iron was quantified using a chemical/colorimetric method while SF was determined using the ferritin quantitative ELISA technique. TSAT was calculated using a formula involving serum iron and total iron binding capacity (TIBC). The latter (TIBC) was also calculated from a formula involving serum iron and unsaturated iron binding capacity (UIBC). UIBC was quantified using the chemical/colorimetric method.

SF estimation was carried out as follows [16]: reagents were left at room temperature and gently mixed, prior to assays; 25 microliters of ferritin standards and samples were introduced with pipettes into appropriately coded wells. Biotin reagent (100 microliters) was added to each well. The plate was shaken for 10-30 seconds. The plate was then covered with a seal (provided by the manufacturer) and incubated for 30 minutes at room temperature (20-25 °C). The liquid was later removed from all wells. The wells were washed three times with 300 microliters of 1X wash buffer and then blotted on absorbance paper. Afterward, 100 microliters of enzyme reagent were added to each well. The plate was covered and incubated for 30 minutes at room temperature. The liquid was subsequently removed from each well. The wells were washed three times with 300 microliters of 1X wash buffer and blotted on absorbance paper; 100 microliters of TMB substrate were added to all wells and incubated for 15 minutes at room temperature. Also, 50 microliters of stop solution were added to all wells. The plate was shaken for 10-20 seconds, to mix to solution. The absorbance on ELISA Reader at 450 nm was taken within 15 minutes, after adding the stopping solution. The optimal densities measured were cross-matched with concentrations of the standards, as provided by the manufacturer. SF measurements were thus recorded in mcg/L.

A known amount of ferrous ions were added to the serum at an alkaline pH. The ferrous ions bind with transferrin at unsaturated iron-binding sites. The additional unbound ferrous ions were measured using the ferrozine reaction. The difference between the amount of ferrous ions added and the unbound ions measured constitutes UIBC. TIBC is equal to the serum iron concentration plus UIBC [17].

TSAT = serum iron ÷ TIBC x 100%.

## Results

Demographic and clinical parameters of participants

The age of the participants ranged from 25 to 78 years with a mean of 53.99 +12.51 years. The majority (n=49, 55.7%) of the participants were middle-aged (45-64 years). There was a slight female preponderance with a total of 47 (53.4%) females and 41 (46.6%) males. The male-to-female ratio was 0.9:1. Most of the participants (n=49, 55.7%) had a tertiary education, followed by 23 (26.1%) with secondary school education and 14 (15.9%) with primary level education. Only two (2.3%) participants had no formal education (Table 1).

**Table 1:** Demographic and clinical characteristics of the study population (N=88) SD: standard deviation

Parameters	Values
Overall age, years, mean +SD	53.93 +12.52
Male, years, mean +SD	54.80 ±10.38
Female, years, mean +SD	53.15 ±14.19
Gender, n (%)	
Male	41 (46.59)
Female	47 (53.41)
Male-to-female ratio	0.9:1
Level of education, n (%)	
No formal education	2 (2.3)
Primary	14 (15.9)
Secondary	23 (26.1)
Tertiary	49 (55.7)

Table 2 shows the clinical parameters of the study participants. They had a mean pulse rate (beats/minute) of 78.45 ±10.19, mean systolic blood pressure (mmHg) of 120.91 ±19.21, and mean diastolic blood pressure (mmHg) of 76.22 ±12.28. The average BMI (kg/m^2^) of the study participants was 27.61 ±6.42; the median (IQR) duration of HF in months was 24 (9.3-60.0), and the majority had had at least one hospital admission: 47.7% had had only one admission while 14.8% had had ≥2 admissions. Of note, 9.1% consumed alcohol while 21.6% consumed herbal medications. The majority (68.2%) had a class II New York Heart Association (NYHA) classification of HF.

**Table 2:** Clinical characteristics of the study population (N=88) SD: standard deviation; HF: heart failure; NYHA: New York Heart Association; IQR: interquartile range

Parameters	Values
Pulse rate, beats/minute, mean ±SD	78.45 +10.19
Systolic blood pressure, mmHg, mean ±SD	120.91 +19.21
Diastolic blood pressure, mmHg, mean ±SD	76.22 +12.28
Body mass index, kg/m^2^,mean ±SD	27.61 +6.42
Duration of HF, months, median (IQR)	24 (9.3-60.0)
Previous hospitalizations, median (IQR)	1 (0-1)
Previous hospitalizations, n (%)	
None	33 (37.5)
1	42 (47.7)
≥2	13 (14.8)
Social habits within 3 months prior to the study, n (%)	
Tobacco use	4 (4.5)
Intake of salty food	4 (4.5)
Alcohol intake	8 (9.1)
Herbal medications	19 (21.6)
No harmful habits	53 (60.2)
NYHA class, n (%)	
II	60 (68.2)
III	15 (17.0)
IV	13 (14.8)
NYHA class, median (IQR)	2 (2-3)

Laboratory measurements

Hematological measurements are presented in Table 3. The mean Hb of the study participants was 12.4 +1.63 g/dl. Anemia was seen in 39 (44.3%) participants. The median ferritin and the mean TSAT values are shown in Table 3. ID was seen in 30 (34.0%) participants: 15 (17.0%) participants had SF <100 mcg/l and 15 (17.0%) participants had SF of 100-299 mcg/L and TSAT <20%. All participants with SF <100 mcg/L had TSAT <20%.

**Table 3:** Laboratory results (N=88) Hb: hemoglobin; MCV: mean corpuscular volume; MCH: mean corpuscular hemoglobin; MCHC: mean corpuscular hemoglobin concentration; WBC: white blood cells; TSAT: transferrin saturation; SF: serum ferritin; IQR: interquartile range

Parameter	Values
Hb, g/dl, mean ±SD	12.4 +1.63
Male	12.54 +1.62
Female	12.28 +1.65
MCV, fl, mean ±SD	86.08 +6.73
MCH, pg, mean ±SD	28.50 +2.26
MCHC, g/dl, mean ±SD	32.94 +1.25
WBC, x 10^9^/l, mean ±SD	5.58 +1.81
SF, mcg/L, median (IQR)	145.76 (107.83-276.01)
TSAT, %, mean ±SD	18.91 +8.69
Incidence of anemia, n (%)	39 (44.3)

Frequency of iron deficiency anemia

**Figure 1:**
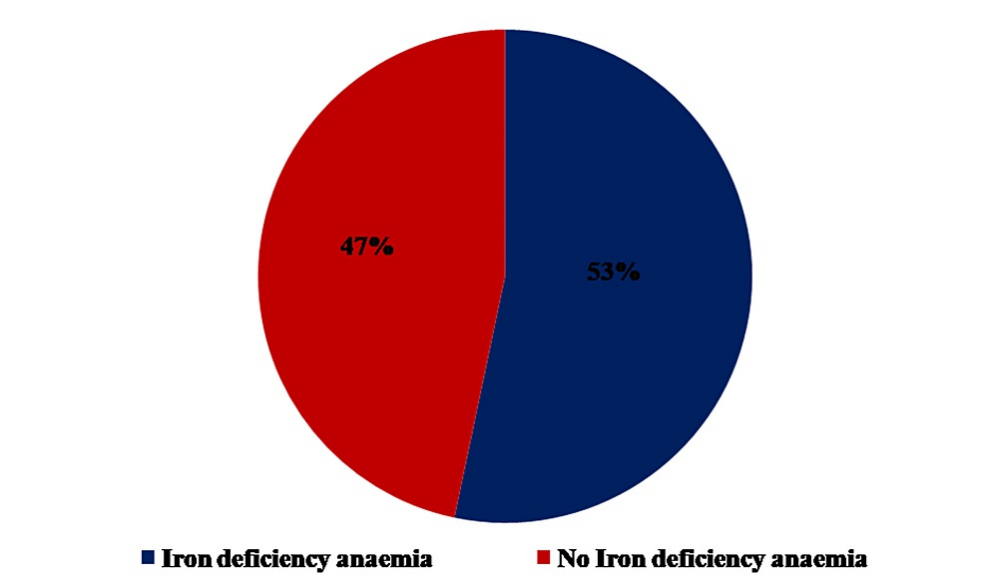
Frequency of iron deficiency anemia

Among the iron-deficient subpopulation (n=30, 34%), only 16 (53.3%) had anemia (Figure 1).

## Discussion

Hb is known to be the major oxygen transporter in humans. Anemia refers to low levels of Hb. Many studies have documented anemia as a reoccurring comorbidity in advanced HF and an independent predictor of poor outcomes in these patients. Anemia has been observed in about 10-50% of patients with chronic HF, and its frequency rises with worsening disease severity [10,18,19]. According to the OPTIME-HF trial, ID is seen in 21% of all cases of HF [20].

Chronic HF with frequent unfavorable outcomes has been linked with ID but the mechanism underlying this association remains unclear [21]. Dysfunction of both the myocardium and skeletal muscles is involved in the pathophysiology of HF. These organs have high energy demands, and their function is dependent on intact iron metabolism [14,22]. The occurrence of this deficiency in chronic HF has been attributed to several factors and the top on the list is inflammation. In chronic HF, the inflammatory processes that occur may result in impaired iron metabolism leading to functional ID.

The diagnosis of ID is traditionally based on the measurement of serum iron levels, ferritin, TIBC, and TSAT (all together referred to as the iron panel), along with hematological evidence of the disease. In some cases, features of the disease may be present clinically. Bone marrow examination for stainable iron used to be considered the gold standard for the diagnosis of ID but is no longer recommended for routine evaluation due to high inter- and intra-observer variability and risk associated with the procedure [23]. However, it may be helpful if there are concerns that a high SF value (>1200 mcg/l) is not a true reflection of the bone marrow iron storage pool [24].

The iron status of a cohort of Nigerian chronic HF patients was assessed in this study. This was performed using one of the following two criteria: SF <100 mcg/L or TSAT <20% (where SF is 100-299 mcg/L). Participants diagnosed using the ferritin-only criterion were termed to have absolute ID while those diagnosed with the ferritin/TSAT criterion were termed to have functional ID [25]. Based on both criteria, the total frequency of ID was 34% (17% for SF-only and 17% for TSAT/SF criterion). This is in line with the report by Jankowska et al. where an incidence of about 37% was documented in 546 patients with chronic HF based on SF of 100-300 mcg/L and TSAT <20% [21].

Adlbrecht et al. found ID (SF <300 mcg/L or TSAT <15%) in 26% of patients with chronic HF, which is similar to the frequency documented in this study [14]. However, the study by Witte et al. showed an ID incidence of only 13%, regardless of LVEF, based on SF <300 mcg/L alone [17]. This difference may be due to the criterion used in the diagnosis, i.e., SF alone. This single criterion may not be as efficient as using both SF and TSAT values in the diagnosis of ID, especially in the setting of chronic inflammation in HF where SF is expected to be elevated.

## Conclusions

In this study, ID was noted to be fairly common in our chronic HF population and was present in about one-third of cases. HF patients with ID should be enrolled in randomized interventional trials in order to address the unmet needs of specific therapy for this patient group. Anemia remains a significant contributing factor to acute decompensation in patients with chronic HF and the correction of ID has been shown to be crucial in preventing morbidity from HF.
